# ARDS Subphenotypes as a Guide to Therapy and Enrollment into Therapeutic Trials: Not So Fast

**DOI:** 10.3390/jcm14176088

**Published:** 2025-08-28

**Authors:** Jesús Villar, Tamas Szakmany, Ognjen Gajic, Diego Casali, Sara Cazorla-Rivero, Alexander S. Niven

**Affiliations:** 1 Ciber de Enfermedades Respiratorias, Instituto de Salud Carlos III, 28029 Madrid, Spain; 2 Li Ka Shing Knowledge Institute, St Michael’s Hospital, Toronto, ON M5B 1W8, Canada; 3 Research Unit, Fundación Canaria Instituto de Investigación Sanitaria de Canarias, Hospital Universitario Dr. Negrín, Barranco de la Ballena s/n, Annex Building, 35019 Las Palmas de Gran Canaria, Spain; scazorla@ull.edu.es; 4 Faculty of Health Sciences, Universidad del Atlántico Medio, Tafira Baja, 35017 Las Palmas de Gran Canaria, Spain; 5 Integrated Hospital Institute, Cleveland Clinic Abu Dhabi, 59 Hamouda Bin Ali Al Dhaheri Street, Abu Dhabi 112412, United Arab Emirates; szakmat@ccad.ae; 6 Division of Pulmonary and Critical Care, Department of Medicine, Mayo Clinic, Rochester 200 First Street SW, Rochester, MN 55905, USA; gajic.ognjen@mayo.edu (O.G.); niven.alexander@mayo.edu (A.S.N.); 7 Cardiothoracic Intensive Care Unit, Cleveland Clinic Abu Dhabi, 59 Hamouda Bin Ali Al Dhaheri Street, Abu Dhabi 112412, United Arab Emirates; casalid@ccad.ae; 8 Department of Medicine, Mayo Clinic, Rochester 200 First Street SW, Rochester, MN 55905, USA

**Keywords:** acute respiratory distress syndrome, ARDS, phenotypes, clinical outcome, biomarkers, patient preferences, randomized clinical trials, personalized medicine, precision medicine

## Abstract

Acute respiratory distress syndrome (ARDS) is a common and highly heterogeneous condition in the critically ill. The association between hyper- and hypo-inflammatory subphenotypes and clinical outcomes has generated significant interest in precise ARDS management. The value of identifying biomarkers to guide treatment and enrollment in future ARDS trials is undisputable. We describe multiple factors complicating the search for subphenotypes and their treatable traits. The observed heterogeneity seen in the clinical course of ARDS is dynamic and influenced by factors beyond lung pathophysiology, including variations in the delivery of best critical care practices, patient comorbidities, and functional status, and patient or family preferences. Current subphenotype definitions lack strong biological plausibility and without clear evidence of benefit from targeted treatments, their use in clinical practice is currently unwarranted.

## 1. Background

Acute respiratory distress syndrome (ARDS) is a heterogeneous syndrome characterized by acute respiratory symptoms resulting from abnormalities of the airways, alveoli, and interstitium, leading to non-cardiogenic pulmonary edema and impaired spontaneous breathing [1,2]. Its overall mortality is about 40% [3]. Since its original definition [4], the clinical characteristics and responses to treatment have varied widely among patients [5]. Advancements in understanding the underlying disease processes associated with ARDS have prompted revisions of its definition and fueled ongoing efforts to identify modifiable or treatable traits to address treatment variability and improve patient outcomes [6].

The current trend towards broadening the diagnostic criteria for ARDS increases the likelihood that more critically ill patients with acute hypoxemic respiratory failure will meet this definition [7,8]. These efforts are further complicated by the dynamic nature of the observed heterogeneity, which is significantly influenced by factors beyond pulmonary pathobiology, including extrapulmonary organ failure, underlying comorbidities and functional status, patient and family preferences, and, in many instances, variations in the consistent delivery of critical care best practices [9,10].

To address this heterogeneity, several authors proposed the introduction of a variety of subphenotypes, with the aim to improve diagnostic efficiency and to introduce more personalized treatment options for this syndrome [11]. Personalized medicine refers to healthcare that is provided based on a medical model that uses the characterization of individuals’ phenotypes and genotypes. This involves offering preventive, diagnostic, curative, rehabilitative, and specific end-of-life care services tailored to each patient. In this concise Perspective, we will examine the use of the theoretical concepts and methodological issues behind these efforts in order to provide an alternative, critical viewpoint and to review what is certain and uncertain about subphenotyping in ARDS and its main etiologies, as well as suggesting possible actionable, forward-thinking research goals.

## 2. General Terms

A phenotype is an observable characteristic that can be measured and described; it is driven by the interaction between genotype and environment [12]. The concept of the ARDS phenotype was first introduced in the literature in 1967 [4]. Some of the observable traits are determined by the patients’ genotype [13], while others are determined by patient and clinical care factors, including age, underlying disease, duration and technique of mechanical ventilation (MV), availability of local health resources, and occurrence of organ dysfunction [12]. The ARDS phenotype could be identified using a combination of biomarkers based on genotypes, proteins, histology, imaging, and clinical features. Of note, a biomarker is not defined by its technological or biological foundation but rather by its reliable, predictive correlation to differential patient responses [14].

A subphenotype refers to a subset of patients within the phenotype who share specific features, while an endotype denotes a subgroup of ARDS patients with common biological mechanisms [12]. The identification of distinct subphenotypes or endotypes in ARDS and ICU patients is a growing field, with the goal of better defining clinical trajectories, outcome prediction, and targeted therapeutic approaches [15]. Notably, no clinical trials have yet been conducted studying the effects of an intervention with stratification based on ARDS phenotype from any etiology [3]; however, it is acknowledged that different causes of ARDS can markedly influence mortality risk [2].

## 3. Heterogeneity of Disease Processes

ARDS is a highly heterogeneous syndromic disease and is therefore difficult to subcategorize using specific phenotypes/subphenotypes/endotypes. Efforts to differentiate between pulmonary and non-pulmonary sources of ARDS have not substantially advanced our understanding of the disease process or improved management. Several causative insults leading to ARDS have been shown to have diverse risk profiles, such as ARDS following transfusion, as well as after trauma or sepsis [16]. While ARDS patients can experience different etiologies, all of them fulfill the current clinical diagnosis of ARDS, which is based on the Berlin criteria [16]. This clinical categorization, as a sensitive tool to identify a life-threatening clinical syndrome, is inherently flawed [2,3,17].

The most recent extension of the ARDS definition [2,5], which further increases sensitivity but reduces specificity by expanding the clinical criteria, will continue to complicate the identification of ARDS subgroups who are at imminent risk of death or who might benefit from therapies targeting specific disease mechanisms. ARDS patients whose symptoms can change spontaneously or who have highly variable features may face risks when participating in trials of potential treatments. If trial participants have a low risk of the outcome that the intervention aims to prevent, the trial—regardless of sample size—may fail to demonstrate the intervention’s efficacy [18]. The improved identification of ARDS patient populations is essential for appropriate patient characterization [19]. Importantly, different ARDS classes may define distinct outcome trajectories even without implying specific underlying mechanisms, particularly in a syndrome lacking a single etiology or definitive biomarker.

## 4. Treatment Effects of Feature Targets in Mechanically Ventilated ARDS Patients

In medicine, the gold standard test for evaluating the effectiveness of clinical interventions is the randomized controlled trial (RCT), which generates an average treatment effect on the population studied rather than an individual effect. Although it is generally accepted that following the publication of RCTs in critically ill patients, physicians apply interventions based on benefits observed in applicable subsets, some interventions have benefited certain patients while harming others. So far, no published RCTs relating to ARDS have adequately addressed the limitations of traditional subgroups analyses. Buell et al. [20] employed a machine learning model to predict the effects of lower versus higher SpO_2_ targets on 28-day mortality for individual patients included in two trials in mechanically ventilated, critically ill adults. In their analysis, applying the SpO_2_ target was predicted to be optimal for each patient, rather than a randomized target, which would have resulted in a reduction in absolute mortality [20]. Their study revealed a highly heterogeneous distribution of predicted individualized treatment effects on 28-day mortality, whereby approximately one-third of patients had an average mortality, one-third reported a higher mortality, and one-third demonstrated a lower mortality [20]. As the authors noted, this heterogeneity may explain the neutral, negative, contradictory, incomplete, or even misleading results observed in multiple RCTs [9,20,21,22]. The heterogeneity of treatment effect (HTE) can be evaluated in large trials; several subphenotyping approaches have focused on this. From the individual patient point of view, however, predicting the individual treatment effect (ITE) in this heterogeneous and rapidly evolving patient population is extremely challenging [23].

## 5. Subphenotyping by Clinical Parameters and Its Problems

Traditional subgroups of ARDS patients were categorized by oxygenation index; additionally, there are certain cut-offs, which seem to be clinically important. For instance, a PaO_2_/FiO_2_ cutoff of 150 mmHg appears to be appropriate, not only for distinguishing between patients with higher or lower mortality but also for guiding therapeutic interventions [24]. A major contribution of this work is the clear distinction between outcomes predicted at the time of ARDS onset, as mandated by the Berlin criteria [16], and outcomes assessed after 24 h of standardized care [24]. Notably, and consistent with secondary analyses of prior RCTs, prone positioning applied specifically to ARDS patients with a PaO_2_/FiO_2_ < 150 mmHg demonstrated a survival benefit [25,26]. Additionally, it appears that only patients who had consistently applied best practice management and remained severely hypoxemic benefitted from ECMO in COVID-19-related ARDS [27]. On the other hand, the HTE could not be demonstrated with this cut-off in contemporary trials involving different PEEP strategies [28].

The pursuit of precise critical care remains challenging because the observed heterogeneity is dynamic rather than static and depends on complex interactions between clinical management and multifaceted patient characteristics. In many cases of ARDS, lung-protective ventilation and targeted treatment of the underlying cause of ARDS, provided within the first 24 h, can alter the clinical trajectory and prevent the progression of severe, persistent hypoxemia. Conversely, deviations from these fundamental management principles may worsen the patient’s condition. Furthermore, patients with ARDS may succumb despite the implementation of optimal ARDS management strategies [10]. For example, severity stratification using the PaO_2_/FiO_2_ ratio (i) can be readily manipulated by routine ICU management, suggesting a change in severity category when no real clinical change has occurred [3,16,29]; (ii) ignores the underlying cause, which can, in broad terms, be linked to prognosis [18]; and (iii) does not account for the presence of age, functional status, co-existing comorbidities, etc., which may confound acute clinical severity assessment and prognostication [2,3,8,29]. We and others have shown that patient risk stratified according to their baseline PaO_2_/FiO_2_ (as mandated by the Berlin criteria) may experience substantial changes within 24 h of routine intensive care management, potentially shifting to a different severity category [3,29]. This normal response to appropriate supportive measures may render initial risk stratification ineffective for trial enrollment, as noted by Petty [30].

The vast majority of subgroup analyses and subphenotyping efforts to characterize ARDS populations have ignored the time variant nature of the disease and have solely based their assessment on a single time-point, usually the recruitment to a clinical trial or, in the case of retrospective database analyses, the first appearance of the clinical features described in the various ARDS definitions. The standardization of the external effects on lung physiology are essential for better lung phenotyping. Villar et al. evaluated 300 ARDS patients to characterize the relationship between clinical and ventilatory status [31]. Drawing inspiration from Forrester et al. [32], the authors defined four clinical subsets using a PaO_2_/FiO_2_ threshold of 150 mmHg and a PEEP level of 10 cm H_2_O, measured both at ARDS diagnosis and 24 h later using a standardized PEEP-FiO_2_ approach. While the overall hospital mortality was 46%, patient classification changed significantly after 24 h of care [31]. The subphenotyping showed a strong association with increasing hospital mortality, as follows: 23% (subset I), 32% (subset II), 44% (subset III), and 60% (subset IV) [31]. Following this process allowed us to identify key factors/subsets that more effectively stratified mortality risk. This subphenotyping approach was validated in an external cohort from another country [33]. Based on the above, we believe that no indices that are currently used for ARDS classification or outcome prediction will be useful for guiding medical treatment unless they are performed and measured in standardized ventilatory settings [29].

It is highly likely that a similar distribution of heterogeneity in predicted individualized treatment effects would be observed when categorizing ventilated ARDS patients based on PaO_2_/FiO_2_ ratio, dead space (VD/VT), endothelial injury, the number of extrapulmonary organ failures, lung imaging, degree of vascular permeability, pulmonary and systemic biological marker levels, or any modifiable or treatable traits [2,6,34,35], with the aim of reducing preventable deaths and healthcare costs [20]. For instance, no RCT has yet been conducted to study the effects of interventions stratified by the number of extrapulmonary organ failures. If mortality (whether at 28 days, 60 days, 90 days, ICU discharge, or hospital discharge) is the primary outcome of interest in ARDS, then numerous potential trial designs exist as we seek to optimize patient care [9]. Some RCTs may demonstrate mortality reductions without fully understanding the mechanisms or clinical markers that are needed to guide real-world bedside application.

## 6. Biomarkers and Inflammatory Subphenotypes

In 2014, Calfee et al. [36] conducted a secondary analysis of a selected population of ARDS patients who had previously been enrolled in two RCTs of the ARDS Network. With the goal of identifying subphenotypes or endotypes with a distinct disease progression and outcomes, Calfee et al. employed latent class analysis—a statistical modeling approach in which multivariable data are reduced to indirectly observed (latent) variables. Initially, the authors considered 27 clinical variables and eight plasma biomarkers, which were obtained at the time of patient enrollment, seeking to group patients with similar clinical and biological profiles. Two distinct subphenotypes/endotypes emerged—hyper-inflammatory and hypo-inflammatory. In that report, the investigators concluded that (i) patients with both severe systemic inflammatory response and septic shock had a higher risk of death than patients with trauma and/or without systemic inflammation, as well as that (ii) patients with mild ARDS may not require high levels of PEEP [8,21]. These findings were neither specific to ARDS nor novel, as a greater disease severity is well-established to correlate with a higher mortality risk. Subsequently, the same research group developed a parsimonious model incorporating three biomarkers, using data from another RCT of the ARDS Network [37]. Although we acknowledge the pathological relevance of identifying biomarkers for targeting therapy and predicting treatment response in future ARDS trials, those studies supporting hyper- and hypo-inflammatory subphenotypes present several important methodological limitations (Box 1), as follows:

1. Blood samples for biomarker analysis were collected within 36–72 h of meeting ARDS diagnostic criteria.
2. Patients with mild ARDS were included, but outcomes stratified by ARDS severity were not reported.
3. External validation in independent cohorts was lacking.
4. Identifying these subphenotypes at the bedside in real time is challenging.
5. As ARDS shares molecular pathways with sepsis and severe pneumonia, and more than 70% of the patients analyzed had sepsis or pneumonia, the hyper/hypo-inflammatory classification may simply reflect the host response to infection rather than to specific ARDS.
6. There were more patients excluded than included in those trials, introducing potential selection bias [36,37,38,39,40,41].
7. Although no specific ventilator management scheme was promulgated, in the simvastatin trial [41], adherence to lung-protective ventilation ranged from only 20 to 39% across study time-points [42] despite protocol recommendations, raising concerns that ventilator-induced lung injury [43] may have contributed to the hyper-inflammatory subphenotype [38].
8. It remains unclear why only two subphenotypes were selected, rather than three or more, as is common in other syndromes [32]. The limited number of patients in some groups and imbalances in clinical features may have influenced this two-class model, creating “pseudo-cohorts”. Due to the potential selection bias for enrollment into the trials, it seems that the number of final subphenotypes was the result of an insufficient number of patients in some of the subpopulations, as well as being related to an imbalance of clinical features in the two “pseudo-cohorts”. In clinical practice, many disease processes are more naturally classified into “hyper–normo–hypo”, “severe–moderate–mild”, or “high–normal–low” categories [31,33].
9. The timing of data collection was not standardized, which may significantly impact patient classification [24].
10. It is not specified whether the analysis used data collected at ARDS onset, at randomization, or after randomization. Furthermore, it is unclear how many patients failed to meet ARDS criteria after randomization.
11. Clinically, it remains uncertain whether these subphenotypes represent treatable traits [2,6].

Box 1 **Limitations of hyper/hypo-inflammatory subphenotypes.**

1. Blood for biomarker measurements were collected within 36–72 h of meeting ARDS criteria.
2. Patients with mild ARDS were included, but outcomes relating to lung severity were not reported.
3. Those subphenotypes were never validated in external cohorts.
4. There is a real challenge in identifying these subphenotypes at the bedside in real time.
5. ARDS shares the same molecular pathways as sepsis or severe pneumonia. More than 70% of patients in the trials used for developing the inflammatory subphenotypes had sepsis or pneumonia.
6. During the screening period of the trials, there were more ARDS patients excluded than included. The hyper-inflammatory class could be a reflection of host response to infections causing ARDS.
7. In some trials, compliance with lung-protective ventilation was reported in less than 40% of patients across all time-points. It is plausible that inflammatory responses to ventilator-induced lung injury were part of the hyper-inflammatory subphenotype.
8. Due to potential selection bias for enrollment into the trials, it seems than the number of final subphenotypes was the result of an insufficient number of patients in some of the subpopulations.
9. The specific time for modeling was not standardized
10. It is unclear if the data for analysis are at ARDS onset, at randomization, or after randomization. Also, it is unknown how many patients did not meet ARDS criteria after randomization.
11. It is unclear whether those subphenotypes are treatable traits.

As a further illustration to the problems mentioned above, we would like to refer to some future studies. An ongoing prospective observational study, the PHIND study (NCT04009330), is evaluating a point-of-care assay to identify hyper- and hypo-inflammatory phenotypes in a projected cohort of 480 patients receiving either MV or high-flow nasal oxygen, with a substantial number of patients reaching ARDS criteria. However, the PHIND study, by design, shares several of the aforementioned limitations. Meanwhile, the PANTHER trial (Precision medicine Adaptive platform Network Trial in Hypoxemic acute Respiratory failure) is investigating pharmacological therapies—including simvastatin—in these subphenotypes. We believe that the use of simvastatin in this RCT is questionable, until the subphenotype that could perhaps benefit from this intervention is better defined [41]. We await further prospective validation data before adopting these subphenotypes to guide ARDS management.

In a recent Delphi study, subphenotyping was identified as a research priority [44]. Despite over a decade of research, no validation studies have yet confirmed the clinical benefit of enrichment strategies based on these subphenotypes, raising the possibility that the findings of Calfee et al. [36] represent a false-positive signal, as previously discussed. We should exercise caution when conducting treatment trials for currently established hyper- and hypo-inflammatory subphenotypes given the potential significant issues, as well as calling for studies to better define ARDS subphenotypes/endotypes. Even with formal validation, applying subphenotyping to RCT designs without a reconfirmation of ARDS diagnosis following a period of standard treatment may be unjustified [45].

**Figure 1:**
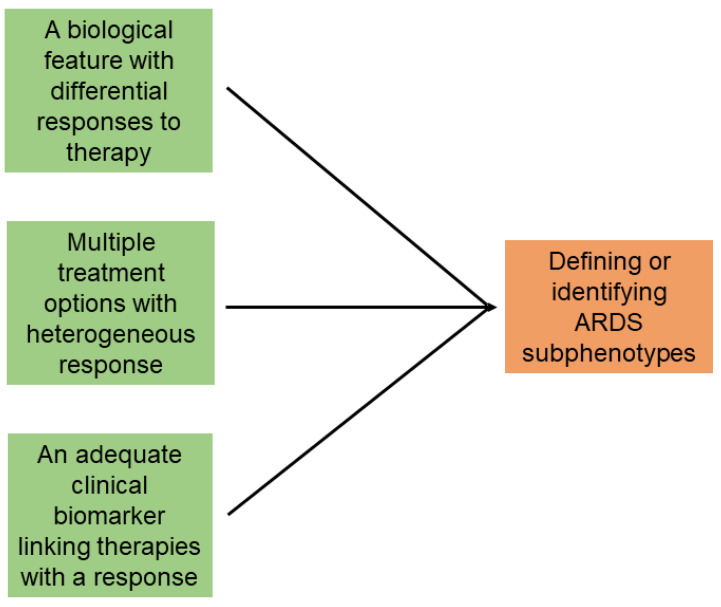
Mandatory conditions for stratified medicine in acute respiratory distress syndrome (ARDS). Source: Trusheim et al. [14].

Importantly, many biomarker studies in critical care—even those rooted in strong physiologic rationale—have failed to demonstrate mortality benefit when rigorously studied [46]. Although the classification of hyper- and hypo-inflammatory subphenotypes is intriguing, its biological significance remains uncertain [6,47,48]. In several more contemporary RCTs, the various inflammatory subphenotypes failed to explain HTE [49]. Pilot studies are needed to determine whether subphenotyping is the optimal approach for ARDS or for acute hypoxemic respiratory failure from any etiology. There is a strong rationale to conduct pragmatic RCTs to address these questions [9]. While clinical biomarkers may potentially identify ARDS subsets that respond to specific therapies, three key elements are necessary for phenotyping definition [14]—(i) a feature capable of mediating differential therapeutic responses; (ii) multiple treatment options with heterogeneous effects; and (iii) an actionable clinical biomarker that links therapies to patients that are likely to respond (Figure 1). Despite the availability of multiple candidate clinical biomarkers [2], major concerns remain regarding the ability to reliably link therapies to ARDS subpopulations.

## 7. Future Directions

It is conceivable that certain ARDS subpopulations may eventually emerge as clinically distinct entities, such as patients who avoid intubation or who respond to non-invasive ventilatory strategies. Several researchers have proposed using prognostic enrichment strategies to enroll better responders in therapeutic RCTs, thereby enhancing trial efficiency and reducing required sample sizes [21,37,50]. The integration of a concise panel of clinical biomarkers with targeted therapies holds promise for improving outcomes, reducing complications and mortality, and avoiding ineffective treatments in relation to ARDS. An “ideal” research agenda and structured trials for ARDS should be systematically explored.

The search for clinically meaningful biomarkers or biomarker panels in ARDS should follow the principles of the SMART framework [51,52], representing criteria that are Specific, Measurable, Attainable, Relevant, and Timely. Goals and targets should be precise and grounded in clinically relevant outcomes, must have some measurements to evaluate and define progress, must be acceptable and reasonable to achieve for clinicians, must relate to expected outcomes with available resources, and must have a realistic timeframe. The closer we align biomarker development with SMART criteria, the more effective and actionable our treatment strategies will become.

A recent report has proposed the implementation of a “Surviving ARDS Campaign” [2] with frequent updates, focused on early diagnosis and management, aimed at improving prevalence data, reducing complication rates, enhancing the detection of comorbidities, addressing treatable traits, and improving survival across all causes of ARDS. Similarly to approaches for sepsis [53], conceptualizing adverse outcomes in ARDS (e.g., ventilator-associated pneumonia, ventilator-induced lung injury, shock, etc.), through the framework of failure to rescue, represents an innovative and provocative approach for quality improvement and ICU education for clinicians, patients, and their families [9,20,53]. These failures may stem, in part, from our incomplete understanding of the underlying pathophysiology of major conditions that precipitate ARDS [9,22].

Notably, in a recent large multicenter cohort study in ICU patients with COVID-19 [10], therapeutic interventions at the time of ICU admission had only a 3% beneficial effect on mortality. Instead, the primary modifiable factors influencing outcomes were related to staffing levels and hospital quality. Thus, hospital-level or ICU-level factors, such as capacity, strain, and overall quality of care, may largely account for variations in ICU mortality rates. In contrast, an individual patient’s risk of death is strongly associated with presenting physiology, demographic characteristics, and preexisting conditions.

Amongst the plausible patient-level modifiable factors, based on the available RCT-level evidence, the lowest hanging research goal is to optimize use of corticosteroids based on the degree of dysregulated inflammation [54]. Further evaluating the HTE and ITE for corticosteroid use might well result in the discovery of various ARDS subphenotypes. At present, however, there is no evidence to support the notion that subphenotype-targeted RCTs in acute hypoxemic respiratory failure or ARDS represent the sole or superior pathway to improving patient care. It is indeed plausible that we are traveling on a methodologically flawed trajectory if we do not step back and try to force a simple dichotomized classification. The loftier goal is to develop a reliable functional test for inflammation, akin to the international normalized ratio or the functional assessment of clotting cascade, with thromboelastography techniques for bleeding/clotting homeostasis. We need to develop something similar for inflammation/immunosuppression, i.e., a test or tests that can monitor the evolving imbalance and rebalance of the immune homeostasis [55]. In parallel, we need to continue and scale up educational and quality improvement interventions that have, so far, most effectively improved the outcomes of critical illnesses [9]. We need to ensure that any biomarkers and clinical features that highlight patient cohorts with different risks and biologically plausible treatable traits are appropriately tested, externally validated, and accessible. Finally, new clinical trials should not continue to ignore important determinants of outcome, such as delays and errors in diagnosis and treatment as well as patient/family preferences for burdensome interventions and prolonged recovery [9].

## Data Availability

When writing the manuscript, the authors did not have access to any special sets of data. As such, the authors cannot provide any special access to datasets that readers might request.
